# Independent external validation of a stroke recurrence score in patients with embolic stroke of undetermined source

**DOI:** 10.1186/s42466-023-00279-z

**Published:** 2023-10-05

**Authors:** Thies Ingwersen, Manuel C. Olma, Eckhard Schlemm, Carola Mayer, Bastian Cheng, Serdar Tütüncü, Paulus Kirchhof, Roland Veltkamp, Joachim Röther, Ulrich Laufs, Darius G. Nabavi, George Ntaios, Matthias Endres, Karl Georg Haeusler, Götz Thomalla

**Affiliations:** 1 Department of Neurology, University Medical Centre Hamburg-Eppendorf (UKE), Hamburg, Germany; 2 Center for Stroke Research Berlin, Charité Universitätsmedizin Berlin, Berlin, Germany; 3 Berlin Institute of Health, BIH, Berlin, Germany; 4 Department of Cardiology, University Heart and Vascular Center Hamburg, Hamburg, Germany; 5 Institute of Cardiovascular Sciences, University of Birmingham, Birmingham, UK; 6 Partner Site Hamburg/Kiel/Lübeck, German Centre for Cardiovascular Research, Hamburg, Germany; 7 Department of Neurology, Alfried Krupp Hospital, Essen, Germany; 8 Department of Brain Sciences, Imperial College London, London, UK; 9 Department of Neurology, Asklepios Hospital Altona, Hamburg, Germany; 10 Department of Cardiology, University Hospital Leipzig, Hamburg, Germany; 11 Department of Neurology, Vivantes Hospital Neukölln, Berlin, Germany; 12 Department of Internal Medicine, School of Health Sciences, University of Thessaly, Larissa, Greece; 13 Partner Site Berlin, German Centre for Neurodegenerative Diseases (DZNE), Berlin, Germany; 14 Excellence Cluster NeuroCure, Berlin, Germany; 15 Department of Neurology with Experimental Neurology, Charité Universitätsmedizin Berlin, Berlin, Germany; 16 Department of Neurology, Universitätsklinikum Würzburg (UKW), Würzburg, Germany

**Keywords:** Ischemic stroke, Embolic stroke of undetermined source, ESUS, Stroke recurrence, External validation, Stroke recurrence score

## Abstract

**Background:**

Embolic stroke of undetermined source (ESUS) accounts for a substantial proportion of ischaemic strokes. A stroke recurrence score has been shown to predict the risk of recurrent stroke in patients with ESUS based on a combination of clinical and imaging features. This study aimed to externally validate the performance of the ESUS recurrence score using data from a randomized controlled trial.

**Methods:**

The validation dataset consisted of eligible stroke patients with available magnetic resonance imaging (MRI) data enrolled in the PreDAFIS sub-study of the MonDAFIS study. The score was calculated using three variables: age (1 point per decade after 35 years), presence of white matter hyperintensities (2 points), and multiterritorial ischaemic stroke (3 points). Patients were assigned to risk groups as described in the original publication. The model was evaluated using standard discrimination and calibration methods.

**Results:**

Of the 1054 patients, 241 (22.9%) were classified as ESUS. Owing to insufficient MRI quality, three patients were excluded, leaving 238 patients (median age 65.5 years [IQR 20.75], 39% female) for analysis. Of these, 30 (13%) patients experienced recurrent ischaemic stroke or transient ischemic attack (TIA) during a follow-up period of 383 patient-years, corresponding to an incidence rate of 7.8 per 100 patient-years (95% CI 5.3–11.2). Patients with an ESUS recurrence score value of ≥ 7 had a 2.46 (hazard ratio (HR), 95% CI 1.02–5.93) times higher risk of stroke recurrence than patients with a score of 0–4. The cumulative probability of stroke recurrence in the low-(0–4), intermediate-(5–6), and high-risk group (≥ 7) was 9%, 13%, and 23%, respectively (log-rank test, χ^2^ = 4.2, *p* = 0.1).

**Conclusions:**

This external validation of a published scoring system supports a threshold of ≥ 7 for identifying ESUS patients at high-risk of stroke recurrence. However, further adjustments may be required to improve the model’s performance in independent cohorts. The use of risk scores may be helpful in guiding extended diagnostics and further trials on secondary prevention in patients with ESUS.

*Trial registration:* Clinical Trials, NCT02204267. Registered 30 July 2014, https://clinicaltrials.gov/ct2/show/NCT02204267.

## Background

After ischaemic stroke, a comprehensive diagnostic evaluation is crucial for initiating appropriate secondary prevention measures [16]. Current international guidelines recommend the use of an antiplatelet drug in non-AF (atrial fibrillation) patients or direct oral anticoagulation in AF patients [2]. Approximately 15–20% of stroke cases are classified as “cryptogenic”, meaning that the aetiology remains unknown despite a comprehensive diagnostic work-up. Embolic stroke is suspected in a significant percentage of cryptogenic cases even in the absence of a proven cardioembolic source [3, 10, 26].

In 2014, the concept of embolic stroke of undetermined source (ESUS) was introduced to categorise these patients and determine the best secondary treatment in randomised controlled trials [11]. Two major trials were conducted in ESUS patients but failed to demonstrate a benefit of DOAC treatment versus aspirin in secondary stroke prevention [3, 5, 12]. This may stem from the heterogeneous nature of strokes summarised under the ESUS label, which encompasses a wide range of possible causes [20, 21].

To advance our understanding of ESUS and develop better treatment strategies, it seems to be essential to further identify ESUS patients with an elevated risk of recurrent stroke. Furthermore, identifying high-risk ESUS patients can help allocate resources more efficiently, as diagnostic evaluation can be resource intensive in these patients.

An integer-based scoring system incorporating both clinical and imaging factors has been proposed by Ntaios et al. and was shown to be useful in the risk stratification of patients with ESUS. In particular, compared to 403 ESUS patients in the lowest tertile (i.e., score of 0–4), 202 patients in the highest tertile (i.e., score of 7–12) had a 4.7 times higher risk of stroke recurrence [19]. Despite these promising results, this score has not been externally validated yet. To address this, the present analysis aimed to externally validate the score’s performance in an independent cohort of patients with ESUS.

## Methods

### Validation cohort

We used data from the *Prediction of Atrial Fibrillation based on Stroke Lesion Characterisation in the MonDAFIS Study* (PreDAFIS) cohort, which is a sub-study of the *Impact of Standardized MONitoring for Detection of Atrial Fibrillation in Ischemic Stroke* (MonDAFIS) study. The study design and participant information have been described in detail previously [7, 8, 22]. Briefly, the MonDAFIS study was an investigator-initiated, randomised, multicentre study sponsored by the Charité – Universitätsmedizin Berlin and funded by Bayer Vital GmbH Germany [8]. The MonDAFIS cohort comprised patients from 38 certified German stroke units who presented with acute ischaemic stroke or transient ischaemic attack with an existing neurological deficit at admission. Patients without known AF at admission were eligible for inclusion and were followed up at 6, 12, and 24 months after enrolment [7]. 3465 patients were allocated to either receive systematic Holter-ECG monitoring (up to 7 days in-hospital) in addition to standard diagnostic care (intervention group), or to receive standard care alone (control group).

The PreDAFIS substudy was initiated to further investigate the role of MRI in identifying patients at high risk of AF and predicting outcomes in these patients. Patients in the MonDAFIS intervention arm with available MRI data were included in the PreDAFIS substudy [22].

Patients in the PreDAFIS cohort were screened according to the ESUS criteria proposed by the Cryptogenic Stroke/ESUS International Working Group [11]. These criteria require that a patient must have a non-lacunar brain infarct with no evidence of extracranial or intracranial atherosclerosis causing ≥ 50% luminal stenosis in the arteries that supply the area of ischaemia, no major-risk cardioembolic source, and no other specific cause of stroke, such as arteritis, dissection, migraine/vasospasm, or drug misuse. Patients classified as having ESUS were eligible for inclusion in the validation dataset.

### Score calculation and risk group allocation

The ESUS recurrence score was calculated using three variables, as previously reported: age (1 point every decade after 35 years), existing white matter hyperintensities (WMH) on MRI (2 points), and acute or chronic multiterritorial ischaemic stroke (3 points) [19].

The severity of WMH was assessed by two experienced neurology residents with training in MRI diagnostics. For this purpose, a modified four-grade version of the Fazekas scale was applied to FLAIR or T2 images [6]. A grade of 0 indicated the absence of deep or periventricular WMH. Grade 1 indicates the presence of periventricular caps, pencil-thin lining of the ventricles, or punctate foci in the deep white matter. Grade 2 was defined as the presence of a smooth periventricular halo or convergence of deep white matter foci in subcortical regions. Grade 3 represents severe confluent periventricular WMH that extends into deep subcortical white matter or large confluent areas. Patients with grade 2 or higher were classified as having WMH.

Multiterritorial stroke is defined as multiple ischaemic lesions affecting at least two of the three territories: left anterior, right anterior, or posterior circulation [22]. Acute stroke lesion locations were evaluated using semi-automatically generated segmentation masks and a published brain atlas that defined arterial territories [22, 27]. FLAIR-weighted magnetic resonance images were re-examined to check for multiterritorial lesion distributions in cases with a known history of ischaemic stroke.

An ESUS recurrence score of 0 to 4 points placed patients in the low-risk group, 5 to 6 points in the intermediate-risk group, and more than 7 points in the high-risk group.

### Statistical analysis and score validation

The score was validated using discrimination and calibration measures. Discrimination is a metric that compares a model's ability to differentiate between patients who have experienced the event in question and those who have not. Calibration is the accuracy with which a model predicts risk. A well-calibrated model predicts the correct probability of an event at all risk levels [24].

#### Descriptive analysis

Descriptive statistics are reported as counts, percentages, medians, and interquartile ranges. To analyse differences between groups for categorical or continuous variables, the Mann–Whitney test or chi-square test was applied, as appropriate for the data type. The risk of stroke recurrence between the groups was compared using Kaplan–Meier cumulative risk estimation [24].

#### Measures of discrimination

Survival curves were generated for the stroke recurrence risk groups using the Kaplan–Meier approach. To quantify the differences between the risk groups, hazard ratios were evaluated using a Cox model [25]. A log-rank test was performed to assess statistical significance. Discrimination was further evaluated by calculating Harrell's index of concordance (C-index) [1, 9].

#### Assessment of general fit and calibration

Calibration slope analysis was used to assess the accuracy of the score. The calibration slope was estimated by fitting a Cox regression model with the risk score as the predictor variable in a Cox model. Furthermore, to check for differences in the regression coefficients for one or more score variables (age, WMH, and multiterritorial infarcts), we fitted a Cox regression with the calculated score as an offset. The resulting model indicated differences between the regression coefficients of the validation and derivation datasets. A coefficient of zero would indicate an optimal specification in our validation dataset. A joint test (ANOVA) was performed to assess the statistical significance of deviations from zero. In addition, this analysis was performed using the published raw coefficients of the derivation model to calculate the prognostic index [1, 25]. The prognostic index (PI) was defined by

$${\text{PI}} = \,0.311 \times {\text{Age}}_{{{\text{decades}}\;{\text{after}}\;35}} + 0.636 \times {\text{WMH}} + 0.903 \times {\text{multiterritorial}}\;{\text{infarcts}}.$$

All analyses were conducted using R version 4.2.2 [23].

## Results

### Baseline patient characteristics

**Fig. 1:**
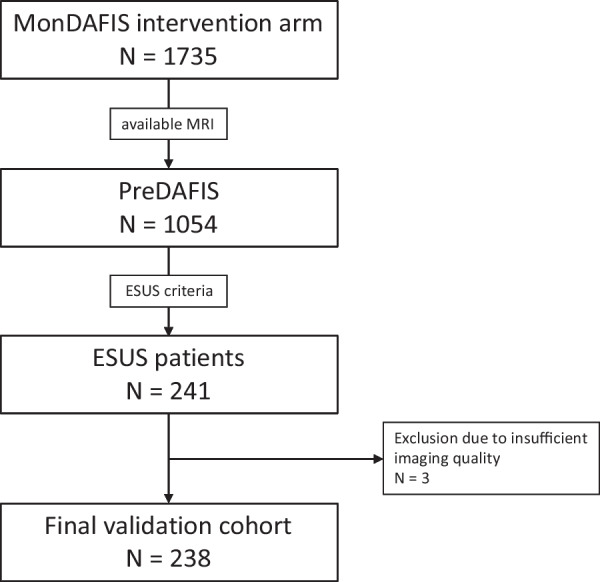
Study flowchart. A total of 1054 patients were included in the PreDAFIS subgroup cohort of which 241 cases were classified as ESUS. After exclusion 238 cases were used for analysis. *ESUS* Embolic stroke of undetermined source

In the PreDAFIS cohort, 241 of 1,054 (22.9%) patients were classified as having ESUS. Three patients were excluded due to insufficient MRI quality. Thus, the validation dataset comprised 238 patients (Fig. 1). Of these, 92 (39%) were female. The median age was 65.5 years (IQR 20.75). The median follow-up time was 721 (IQR 83) days, corresponding to a total follow-up time of 382.5 patient-years. 30 (13%) patients experienced recurrent stroke or TIA. This corresponds to 7.8 (95% CI 5.3–11.2) recurrent strokes per 100 patient-years. Additional baseline characteristics are summarised in Table 1.

**Table 1:** Baseline characteristics comparing patients with and without recurrent stroke

	Total	Patients without recurrent stroke/TIA	Patients with recurrent stroke/TIA	*p* values
	N = 238	N = 208	N = 30	
Age, years (IQR)	65.5 (20.75)	65 (21)	67.5 (17.25)	0.524
Sex, female (%)	92 (39)	83 (40)	9 (30)	0.328
NIHSS (IQR)	2 (3)	2 (3)	2.5 (3)	0.876
Prior stroke/TIA (%)	45 (19)	32 (15)	13 (43)	**0.002**
Hypertension (%)	173 (73)	148 (71)	25 (83)	0.193
Diabetes (%)	59 (25)	49 (24)	10 (33)	0.261
Dyslipidaemia (%)	113 (47)	97 (47)	16 (53)	0.574
Coronary artery disease (%)	25 (11)	21 (10)	4 (13)	0.743
Atrial fibrillation during follow-up (%)	19 (8)	14 (7)	5 (17)	0.061
WMH, Fazekas ≥ 2 (%)	146 (61)	123 (59)	23 (77)	0.069
Fazekas score (IQR)	1 (1)	2 (1)	2 (0)	0.201
WMH volume, ml (IQR)	5.9 (11.3)	5.5 (11.5)	6.9 (6.1)	0.371
Multiterritorial infarcts (%)	22 (9)	14 (7)	8 (27)	**0.001**
Stroke recurrence score (IQR)	5 (4)	5 (4)	6 (3.5)	**0.014**

Data reported as number (percentage) or median (IQR interquartile range)

*P* < 0.05 are presented in bold

*TIA* transient ischemic attack, *NIHSS* National Institutes of Health Stroke Scale, *WMH* white matter hyperintensities

### Score validation

**Fig. 2:**
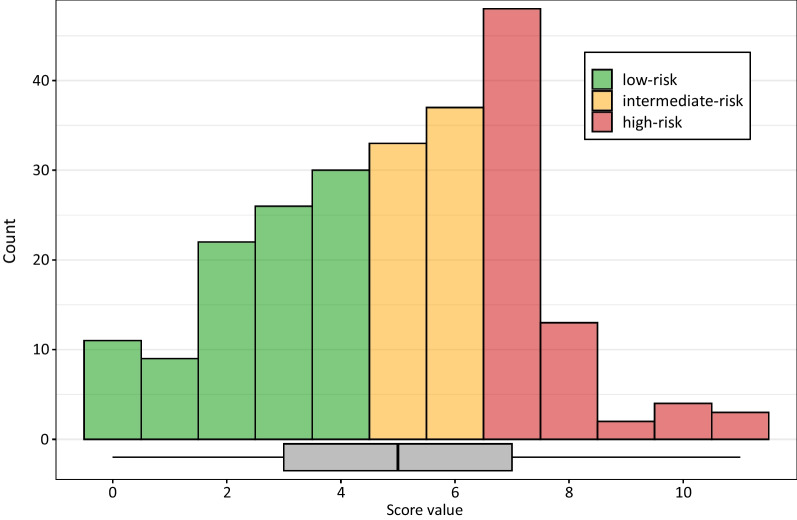
Histogram and boxplot displaying score distribution. Bars are coloured according to the risk group affiliation. A boxplot shows a median score value at 5 points (interquartile range 4)

The median score value in the validation cohort was 5 (IQR 4), with 98 (41%) patients assigned to the low-risk group and 70 (29%) patients each assigned to the intermediate- or high-risk group (Fig. 2). The median scores for the low-, intermediate-, and high-risk group were 3 (IQR 2), 6 (IQR 1), and 7 (IQR 1), respectively. The rate of stroke recurrence was 4.8 (2.1–9.5) per 100 patient-years in the low-risk group, 8.0 (3.7–15.2) in the intermediate-risk group, and 12.5 (6.7–21.4) in the high-risk group. The cumulative probability of recurrent stroke was 8.6% (4–15%) in the low-risk group, 13% (6–22%) in the intermediate-risk group, and 23% (12–36%) in the high-risk group (log-rank test: χ^2^ = 4.2, *p* = 0.1).

**Fig. 3:**
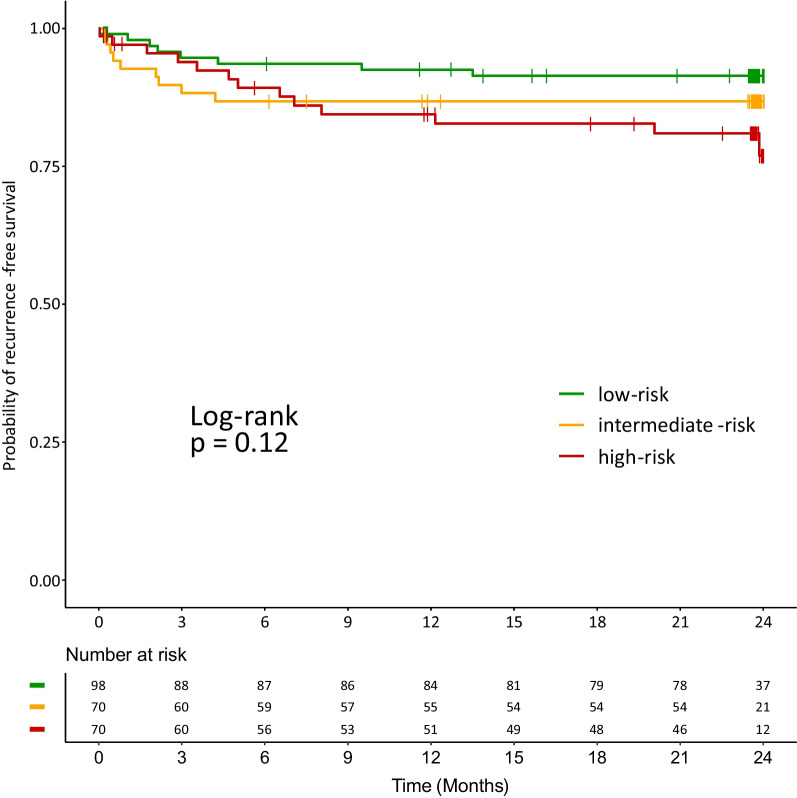
Kaplan–Meier curves for cumulative probabilities of stroke recurrence-free survival across risk groups. A log-rank-test was not statistically significant (*p* = 0.12)

Figure 3 shows the Kaplan–Meier curves for stroke-free survival based on the assigned risk group with a log-rank test of *p* = 0.12. In Cox regression analysis, high-risk patients had a 2.46 (1.02–5.93, *p* = 0.046) times increased risk of stroke recurrence compared to low-risk patients. Discrimination between the low- and intermediate-risk groups did not seem to be maintained, as the hazard ratios did not show a statistically significant increase in the Cox model (Table 2). Harrell's C-index was 0.59.

**Table 2:** Hazard ratios for recurrent stroke probability by risk groups in a Cox model

Variable	HR (95% CI)	*p* values
Intermediate- versus low-risk	1.63 (0.63–4.24)	0.312
High- versus intermediate risk	1.50 (0.64–3.52)	0.348
High- versus low-risk	2.46 (1.02–5.93)	**0.046**

Risk groups were defined by score value as described in the text

*P* < 0.05 are presented in bold

*HR* hazard ratio, CI confidence interval

The Cox regression model for the score showed a slope of 0.22 (standard error [SE] 0.08), which was significantly different from 1 (*p* = 0.007). Similar results were obtained when the slope was calculated using the prognostic index (0.70 [SE 0.26, *p* = 0.007]). Table 3 presents the results of the Cox model analyses using the individual score items as covariates, with the calculated score or the prognostic index as an offset. Using the integer score as an offset in the first model, the coefficients of the three score variables were statistically significantly different from zero (joint test: χ^2^ = 120, *p* < 0.001, Table 3).

**Table 3:** Cox regression models on the score variables with the integer score value or the prognostic index as an offset

Variable	Beta (SE)	HR (95% CI)	*p* values
*Integer score value as an offset Joint test (ANOVA): χ*^2^ = 120, *p* < 0.001			
Age_decades after 35_	− 1.02 (0.15)	0.36 (0.27–0.48)	< 0.001
WMH	− 1.37 (0.49)	0.26 (0.10–0.67)	0.006
Multiterritorial infarcts	− 1.56 (0.42)	0.21 (0.09–0.48)	< 0.001
*Prognostic index as an offset Joint test (ANOVA): χ*^2^ = 7.34, *p* = 0.06			
Age_decades after 35_	− 0.33 (0.15)	0.72 (0.54–0.96)	0.024
WMH	− 0.002 (0.49)	1.00 (0.38–2.62)	0.997
Multiterritorial infarcts	0.53 (0.42)	1.70 (0.74–3.91)	0.219

The prognostic index is calculated with the unrounded coefficients from the original model. A coefficient of zero indicates an optimal fit. A joint test (ANOVA) was conducted to evaluate the significance of deviations from zero

*SE* standard error, HR, hazard ratio, ANOVA analysis of variance, WMH white matter hyperintensities

However, the joint test was not statistically significant when using the prognostic index as an offset, and the coefficients for WMH and multiterritorial infarcts were not statistically significantly different from zero. A small difference in the Age_decades after 35_-variable was detected (beta: − 0.33 [SE 0.1451], *p* = 0.024, Table 3).

**Fig. 4:**
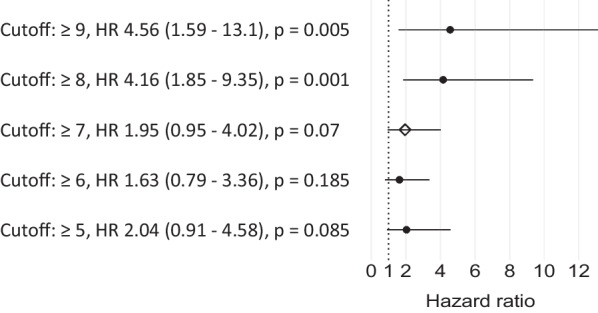
Exploratory analysis of hazard ratios of different score cut-offs. The previously suggested threshold of ≥ 7 points showed a trend towards statistical significance. Patients with a score of ≥ 8 points had a 4.16 times increased risk of stroke recurrence (95% CI 1.85–9.35, *p* = 0.001). *HR* hazard ratio, *CI* Confidence interval

There was a trend of higher risk of stroke recurrence in patients with a score of ≥ 7 compared to those with a score of ≤ 6 (HR 1.95, 0.95–4.02, *p* = 0.07). In an exploratory analysis, we found that the optimal cut-off was 8 points in our cohort (HR 4.16, 1.85–9.35, *p* = 0.001, Fig. 4).

## Discussion

The aim of this analysis was to provide independent external validation of the performance of a score previously proposed to predict recurrent stroke in patients with ESUS [19]. Our analysis in a cohort from a randomised trial confirmed the reliability of the previously suggested threshold of ≥ 7 points to identify ESUS patients at high risk for recurrent stroke [19]. Patients with a score of ≥ 7 were more than twice as likely to have another stroke than patients with a score ≤ 4 points. In an exploratory analysis, an increase of only one point to a cut-off of 8 points resulted in an even better performance in differentiating between high-risk and low-medium risk patients in our validation cohort. These results support the use of the an ESUS recurrence score in clinical practice for the identification of high-risk ESUS patients, especially given the applicability of the score, as it requires only three variables: age, WMH, and multiterritorial infarct.

One may argue that a C-index of 0.59 is only moderate and, therefore, insufficient for accurately estimating the risk of stroke recurrence in a specific patient. However, the purpose of this score is not to provide an accurate risk estimate. Rather, its aim is to identify an ESUS subgroup that is at high risk for stroke recurrence. A similar approach was adopted with the CHA_2_DS_2_-VASc score, which, despite having a similarly moderate C-statistic (0.60) in its derivation cohort [17], is recommended by guidelines for stratifying stroke risk in AF patients [14, 15]. The CHA_2_DS_2_-VASc score is not used clinically to determine the exact stroke risk of a particular AF patient but rather to identify a subgroup at low stroke risk. Similarly, the ESUS recurrence score identifies an ESUS subgroup (i.e., patients with a score of ≥ 7) that has a higher risk of stroke recurrence compared to patients with a lower score.

We calculated a wide variety of additional validation measures, including those of general fit and discrimination. A calibration slope of 0.22 suggests poorer discrimination in our cohort than in the derivation cohort. One explanation why the slope may differ from 1 in our cohort is that there are differences in the regression coefficients for the score variables in our validation dataset compared to the derivation dataset. We checked for model misspecifications in two Cox regression models. In the first model, the integer score is incorporated as an offset, that is, the coefficient of the score is set to 1. In such a model, the coefficients of the score variables should be close to zero given a perfect fit. However, in this cohort, we observed that all coefficients were statistically significantly different from zero when analysed using the integer score (Table 3).

During the development of the score in the original cohort [19], it was derived as an integer-based model by dividing each coefficient of the derivation model by the lowest coefficient and rounding to the nearest integer. To eliminate possible rounding errors, we directly used the coefficients from the derivation model and calculated the prognostic index for each patient. In the second model, this prognostic index was added as an offset as in the first model. In our validation dataset, we found no overall evidence of a lack of fit of the prognostic index since the joint test of the covariates was not statistically significant (χ^2^ = 7.34, *p* = 0.06). However, the statistically significant difference to zero of the Age_decades after 35_-coefficient indicates substantial differences in the association between age and recurrent stroke in the validation and derivation datasets (Table 3). Indeed, there was no statistically significant age difference in patients with or without recurrent stroke in our cohort (Table 1) as opposed to the derivation cohort (median age without and with recurrent stroke in the derivation dataset was 63.7 vs 70.3 years [*p* < 0.001], [19]).

Some of the differences analysed in these two models might be partly explained by differences in the definitions or measurements of the score variables. Due to its availability in our dataset, WMH was defined using the Fazekas scale with a cut-off of ≥ 2 points in FLAIR or T2 weighted magnetic resonance images. This was slightly different from the definition of WMH used in the original study: WMH was defined as patchy or diffuse areas of hypodensity in computer tomography or hyperintensity in magnetic resonance imaging. Further differences may stem from differences in the clinical characteristics among the cohorts. Finally, the moderate number of patients and recurrent strokes in our cohort resulted in a reduction in statistical power and an increased likelihood of type II errors. Previous proposals for a minimum sample size in external validation studies have suggested at least 100 events; however, these were based on a single simulation study [1, 28].

It is noteworthy that the score’s ability to differentiate between high-risk (≥ 7 points) and low-intermediate-risk patients (≤ 6 points) was only moderate in our derivation cohort (Fig. 4). Using a more conservative cut-off value of ≥ 8 points, the high-risk group had a four times increased risk of stroke recurrence compared to patients with ≤ 7 points. It is likely that this shift in results after changing the cut-off by only one point is due to a statistical uncertainty induced by the relatively small sample size as well. However, the score’s ability to differentiate between high-risk and low-risk patients is arguably sufficient for the intended use cases of the score.

Altogether, we consider our cohort as a suitable dataset for the external validation of this ESUS recurrence risk score. Patients in the MonDAFIS study were followed for 2 years, resulting in a thorough examination of these patients. The baseline characteristics of our cohort were mostly similar to those of the derivation cohort. However, we observed a higher stroke recurrence rate of 7.8 per 100 patient-years (vs 3.7 per 100 patient years in the derivation cohort). This might in part be explained by patients being slightly older and more affected by certain cardiovascular risk factors. On the other hand, ESUS patients in our cohort appeared to be less severely affected by the index stroke, as reflected by a lower NIHSS score at admission of 2 points compared with a NIHSS score of 6 points in the derivation cohort [19].

Two large randomised clinical trials, the NAVIGATE ESUS trial [12] and the RESPECT-ESUS trial [5], were recently conducted to evaluate the efficacy of oral anticoagulants versus aspirin in reducing recurrent strokes in patients with ESUS. Although neither trial showed a significant reduction in recurrent strokes in the anticoagulation arm compared with the aspirin arm, subsequent subgroup studies identified groups that benefited from anticoagulation compared with aspirin, such as patients with left ventricular dysfunction [18], patients with an enlarged left atrial diameter [13], and patients aged ≥ 75 years [4]. As the ESUS recurrence score identifies patients at an increased risk of stroke recurrence, it may be useful for further subgroup analyses. It could be hypothesised that high-risk patients would benefit from anticoagulation therapy as well. Furthermore, including cardiac parameters, such as left ventricular dysfunction or atrial volume, in the score calculation might improve the ability of the score to identify high-risk patients who benefit from oral anticoagulation.

## Conclusions

In conclusion, we provide a state-of-the-art, independent, external validation analysis for an easily applicable score that identifies patients with ESUS at a high risk of stroke recurrence. Our findings support the utility of this score in identifying high-risk patients, which may be useful in designing future secondary prevention studies in patients with ESUS. Furthermore, the score might find its way into clinical practice to improve the allocation of resources in the diagnostic work-up of patients with ESUS, especially given its applicability, as it is calculated from only three easily assessed parameters.

## Data Availability

Deidentified participant data with corresponding data dictionary of the data underlying the current manuscript will be made available upon reasonable request. Data will be shared to external researchers for scientific non‑commercial purposes after approval of the proposal by the MonDAFIS steering board including a signed data access agreement.

## Abbreviations

AF: Atrial fibrillation
ANOVA: Analysis of variance
CHA2DS2-VASc: Congestive heart failure, hypertension, age ≥ 75, diabetes mellitus, stroke/TIA/thromboembolism, vascular disease, age 65–74, sex category
CI: Confidence interval
DOAC: Direct oral anticoagulant
ECG: Electrocardiogram
ESUS: Embolic stroke of undetermined source
FLAIR: Fluid-attenuated inversion recovery
HR: Hazard ratio
IQR: Interquartile range
MonDAFIS: Impact of Standardized MONitoring for Detection of Atrial Fibrillation in Ischemic Stroke
MRI: Magnetic resonance imaging
NAVIGATE-ESUS: New Approach Rivaroxaban Inhibition of Factor Xa in a Global Trial versus ASA to Prevent Embolism in Embolic Stroke of Undetermined Source
NIHSS: National Institutes of Health Stroke Scale
PI: Prognostic Index
PreDAFIS: Prediction of Atrial Fibrillation based on Stroke Lesion Characterisation in the MonDAFIS Study
RESPECT-ESUS: Randomized, Double-Blind, Evaluation in Secondary Stroke Prevention Comparing the Efficacy and Safety of the Oral Thrombin Inhibitor Dabigatran Etexilate versus Acetylsalicylic Acid in Patients with Embolic Stroke of Undetermined Source
SE: Standard error
TIA: Transient ischemic attack
WMH: White matter hyperintensities
